# Supplementary material for: Structural basis for the interaction between the Drosophila RTK Sevenless (dROS1) and the GPCR BOSS
Source: Nat Commun. 2025 Jan 18;16:808. doi: 10.1038/s41467-025-55943-6 (PMC11743138; doi:10.1038/s41467-025-55943-6)
Supplement: Supplementary file 1 — Supplementary Information [file 41467_2025_55943_MOESM1_ESM.pdf]

Supplementary Information

Title: Structural Basis for the interaction between the *Drosophila* RTK Sevenless (dROS1) and the GPCR BOSS

Jianan Zhang[1,2,3], Yuko Tsutsui[1,2,3], Hengyi Li[1,2,3], Tongqing Li[1,2], Yueyue Wang[2], Salma Laraki[1,2], Sofia Alarcon-Frias[1,2], Steven E. Stayrook[1,2], Daryl E. Klein[1,2,*]

Affiliations:

[1] Department of Pharmacology, Yale University School of Medicine, New Haven, CT 06520, USA

[2] Yale Cancer Biology Institute, Yale University, West Haven, CT 06516, USA

[3] These authors contributed equally

***Correspondence:** daryl.klein@yale.edu

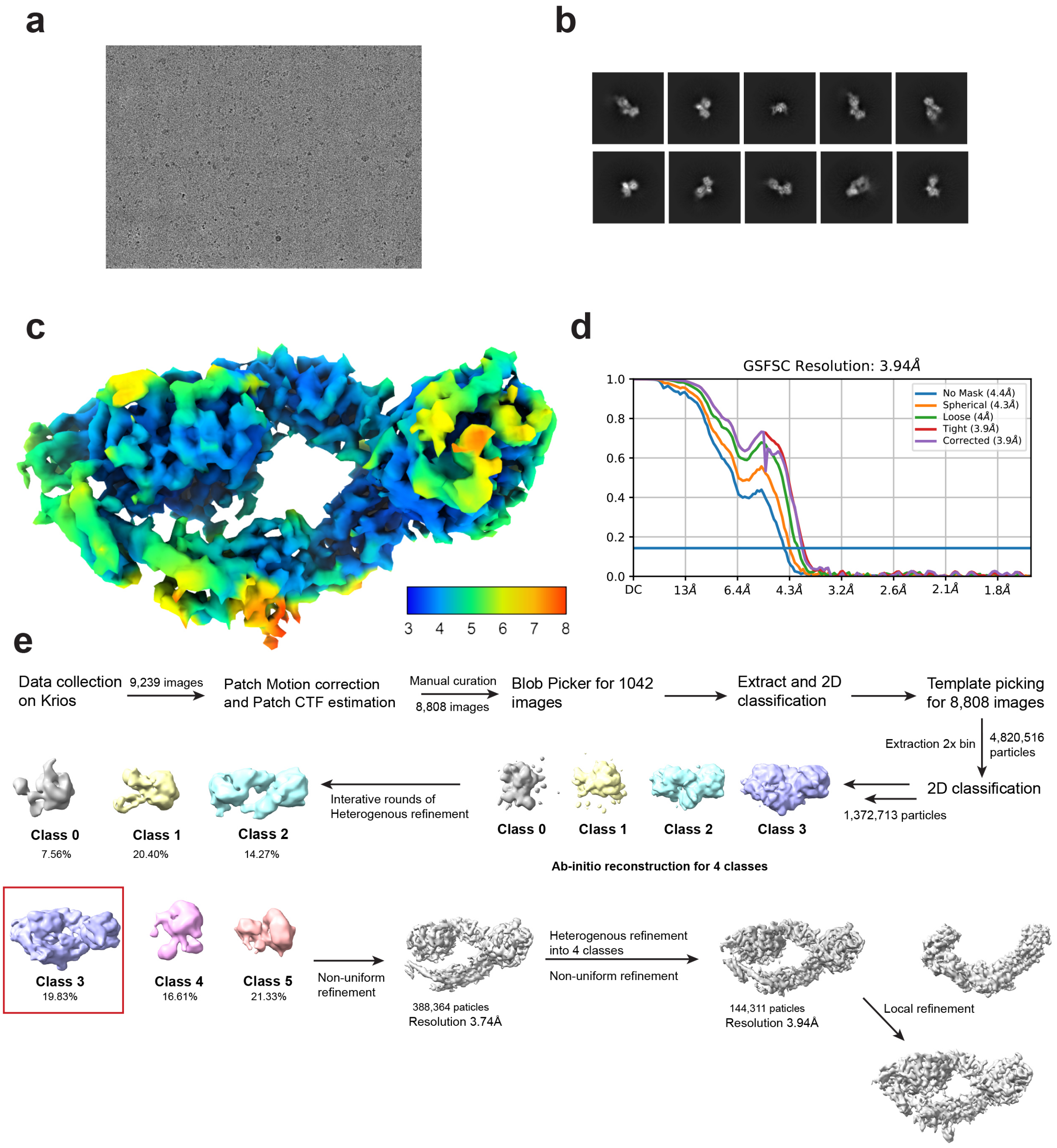

Supplementary Fig. 1 | CryoEM map quality, resolution estimate and workflow of data analysis. a, SDS-PAGE analysis of protein purification and a representative CryoEM micrograph of dROS1. **b,** Selected 2D class average. **c,** 3D reconstructed map colored with local estimated

resolution in angstrom (Å). **d,** Gold-standard FSC curve. **e,** Workflow of data analysis using CryoSPARC4.3.

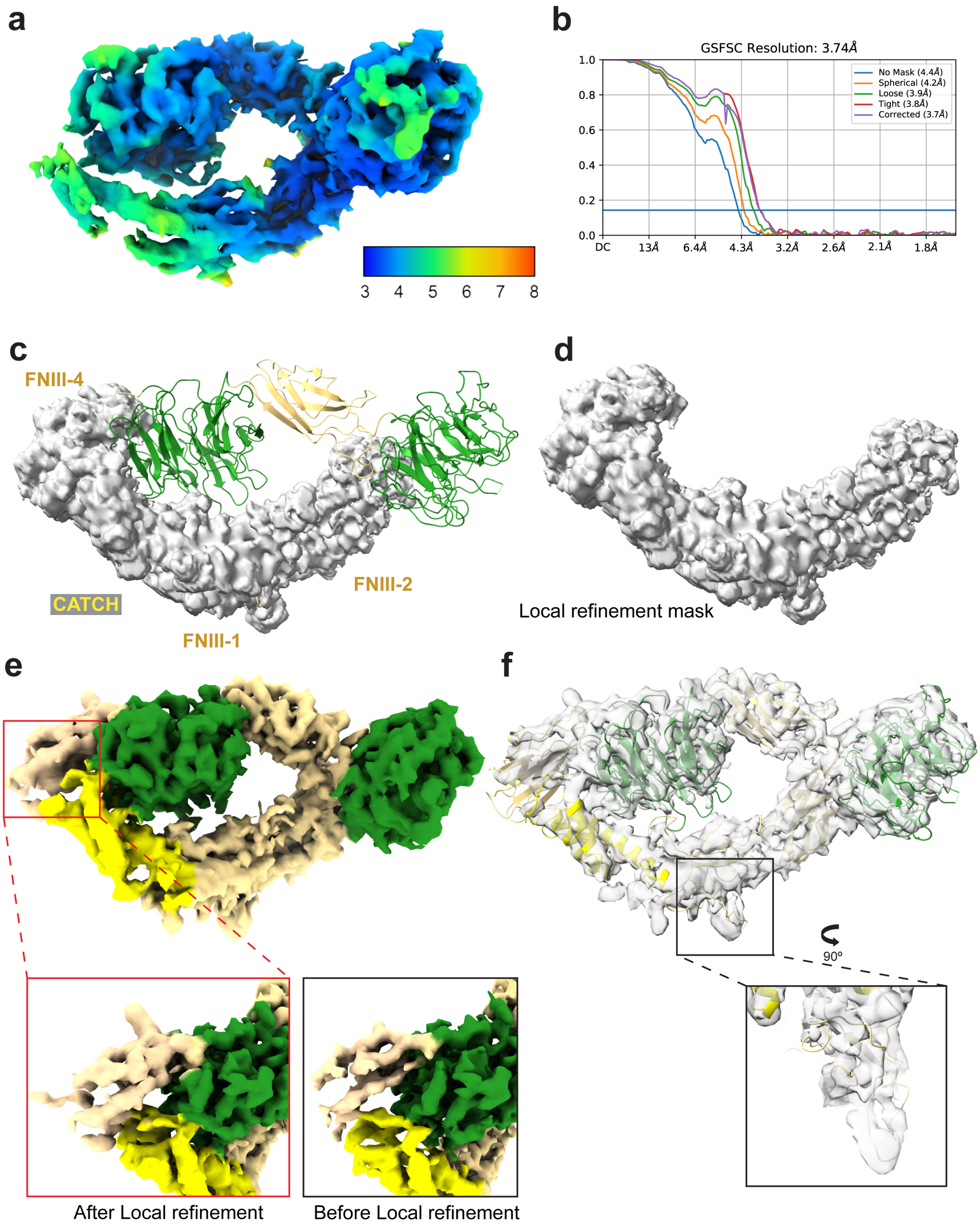

a
3 4 5 6 7 8
b
GSFSC Resolution: 3.74Å
No Mask (4.4Å)
Spherical (4.2Å)
Loose (3.9Å)
Tight (3.8Å)
Corrected (3.7Å)
1.0
0.8
0.6
0.4
0.2
0.0
DC 13Å 6.4Å 4.3Å 3.2Å 2.6Å 2.1Å 1.8Å
c
FNIII-4
CATCH
FNIII-2
FNIII-1
d
Local refinement mask
e
After Local refinement
Before Local refinement
f
90º

Supplementary Fig. 2 | CryoEM map with 3.74 Å and local refinement. a, 3D reconstructed map of 3.74 Å resolution from 388,364 particles. Map was resolved with higher resolution but less density near the CATCH domain, thus this map was only used to aid model building. **b,** Gold-standard FSC curve of the 3.74 Å map. **c,** and **d,** Mask creation for local refinement covering the CATCH domain and the FNIII-4. Additional coverage of FNIII-1 and FNIII-2 was used to reach enough volume for local refinement. **e,** Density map after local refinement using CryoSPARC. More density was observed in FNIII-4. However, no side-chain density was found in the capping region. The display level for maps before and after refinement is both at 0.0804. **f,** More density was observed in the loop region within FNIII-1.

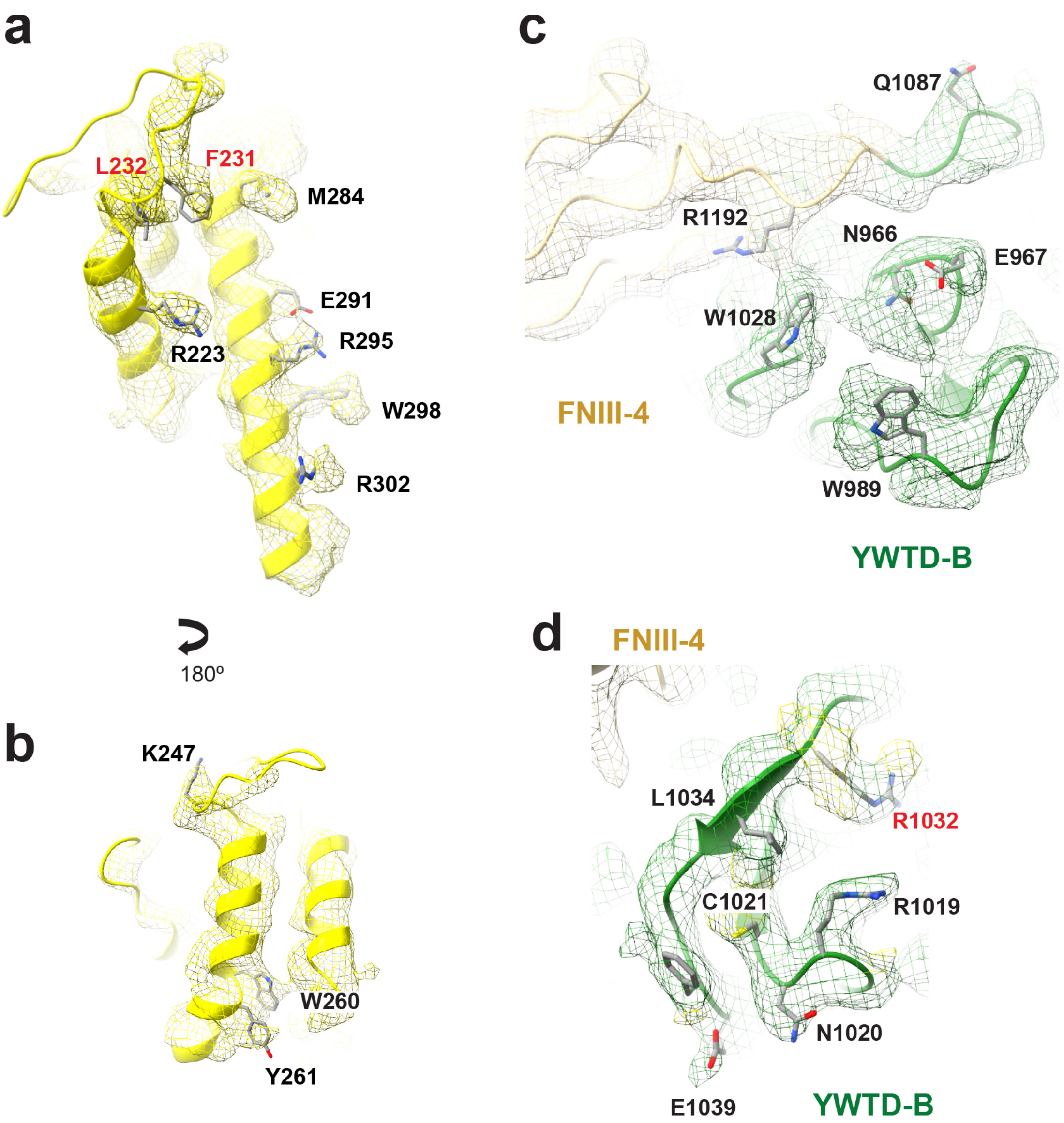

Supplementary Fig. 3 | Local density maps of CATCH, YWTD-B, and FNIII-4 domains. a and **b,** EM density maps of the N-terminal cysteine rich (CATCH) domain. **c and d**, YWTD-B and FNIII-4. Residues in red L232, F231 and R1032 participate in the capping interaction (as shown in **Fig. 2**).

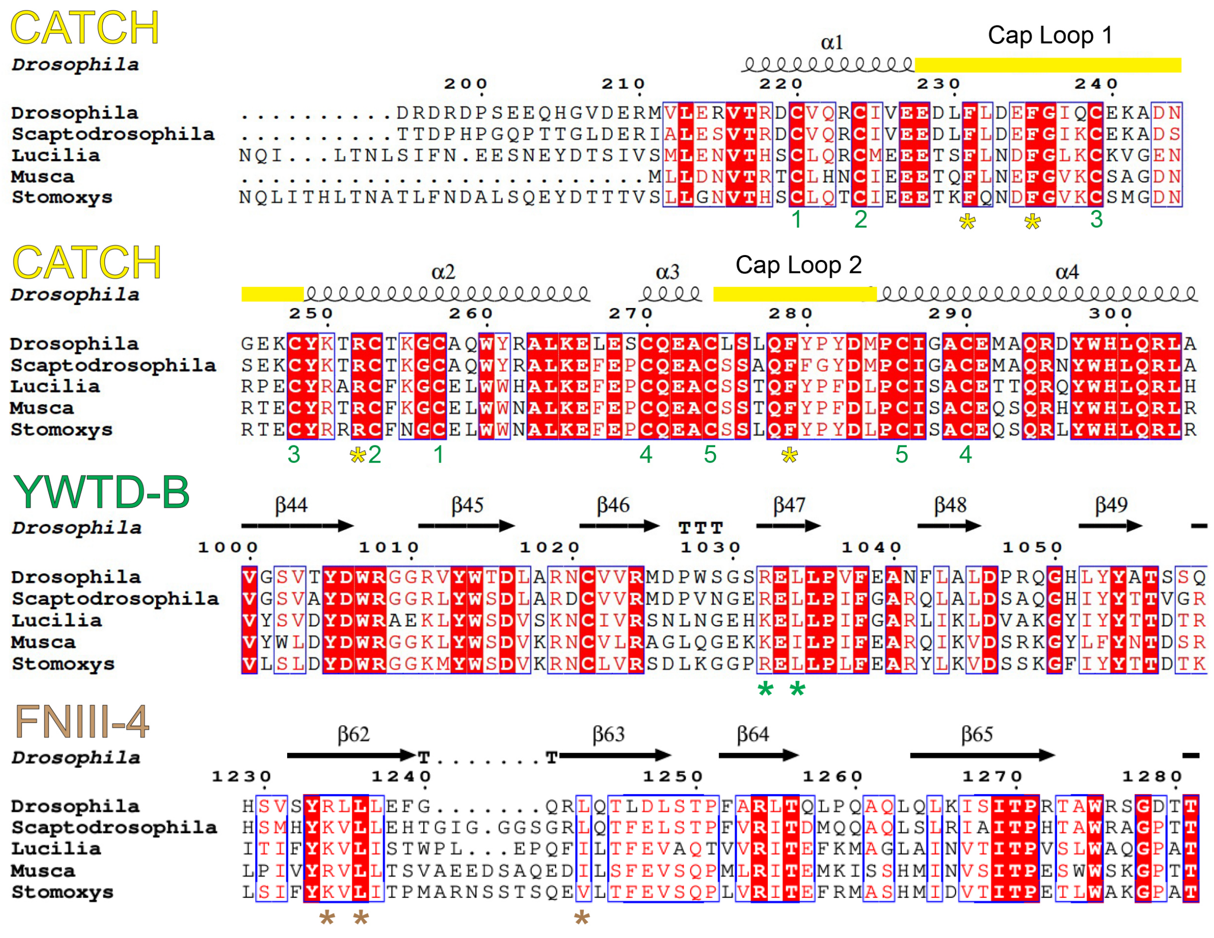

Supplementary Fig. 4 | Sequence alignment of N-term Cysteine rich region through insect species. Residues that are involved in capping interaction were conserved across different insect species. Residues important for the interaction are marked with an asterisk. Alignment was performed using pairwise sequence alignment (EMBL-EBI) and ESpript3.0.

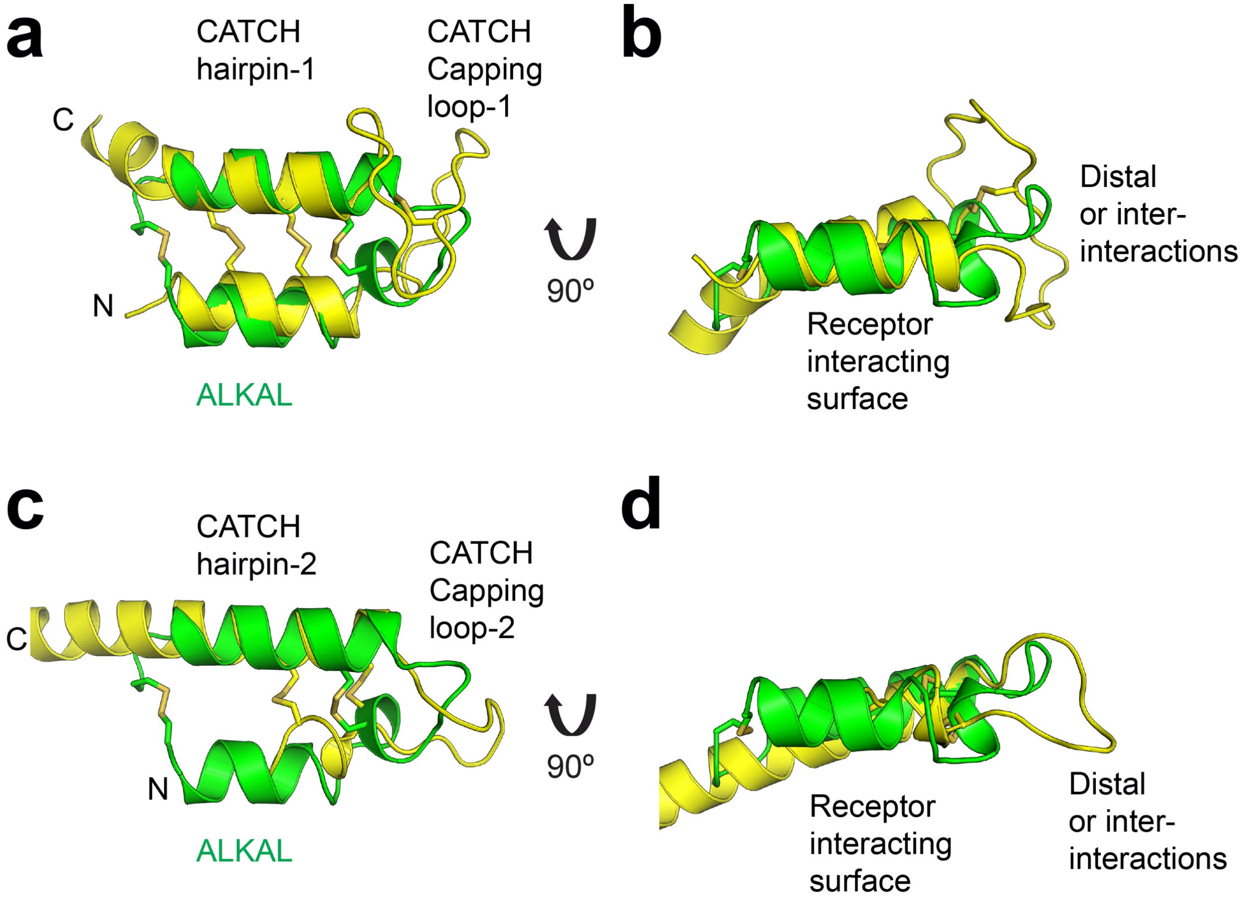

Supplementary Fig. 5 | Structural alignment of CATCH domain with ALKAL. a, Superimposition of doubly disulfide bonded helical hairpin-1 of the CATCH domain (yellow, N-terminal cysteine-rich domain of dROS1) with ALKAL (green). **b,** Rotation from panel **a**. Both ALKAL and CATCH hairpin-1 use the same surface for interactions. The loop region is used for distal receptor interactions in dROS1, and for binding a second receptor for ALKAL. **c,** Alignment as in panel **a** but with the second hairpin of the CATCH domain. **d,** Both ALKAL and CATCH hairpin-2 use the same surface for interactions.

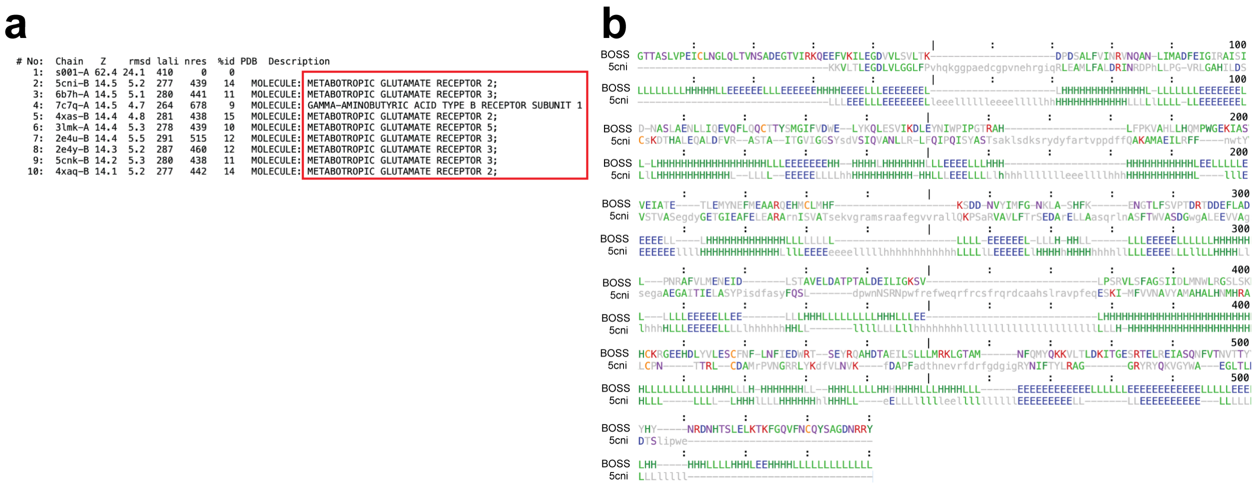

Supplementary Fig. 6 | Structural and sequence alignments of BOSS with mGluR. a, Summary of 3D structural comparison with BOSS' ECR as a template using the Dali server[1,2]. Results with the highest Z-scores fall into mGluRs (metabolic glutamate receptors). **b,** Amino acid sequence and secondary structure alignment are shown.

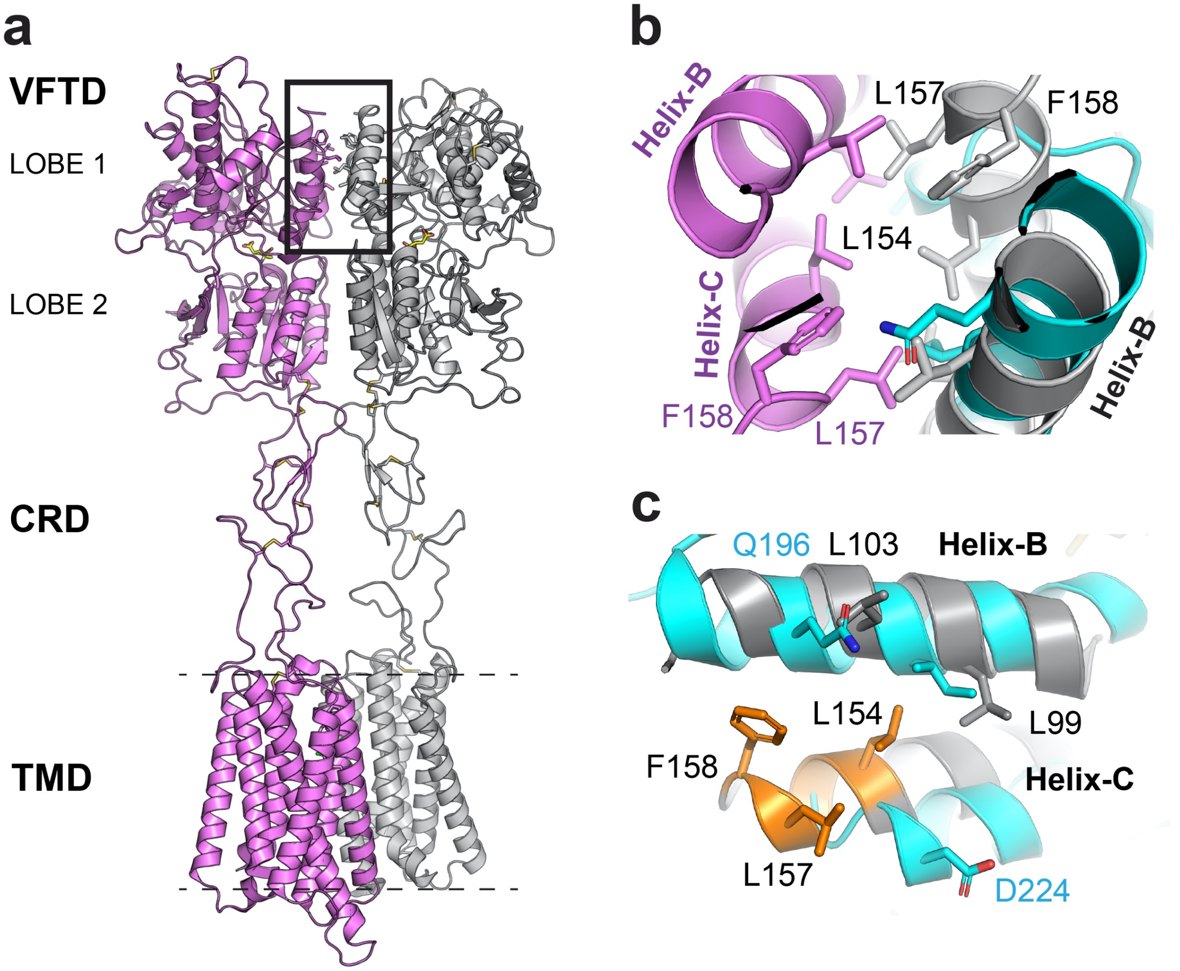

Supplementary Fig. 7 | BOSS comparison to mGluR-2 and lack of dimer interface.

a, Structure of full length, homodimeric, agonist bound mGluR-2 (PDB: 7E9G)[3]. One protomer colored purple and one colored gray. A cysteine rich domain (CRD) stem separates the VFTD from the transmembrane domain (TMD). **b,** A close up view of the boxed region in panel **a**. The ECR of BOSS (cyan) is aligned to a single protomer. The dimerization of mGluR-2 is directed by Helices B and C of Lobe 1. Multiple hydrophobic residues pack at the interface, including: L154, L157 and F158. BOSS (cyan) aligned to Lobe 1 of a single mGluR-2. **c,** Details of the alignment of BOSS (cyan) to Lobe 1 of mGluR-2 (gray and orange). BOSS does not maintain the

hydrophobic character of the dimeric interface. Helix-C of BOSS is smaller and does not retain the critical residues from 154-158 (colored orange).

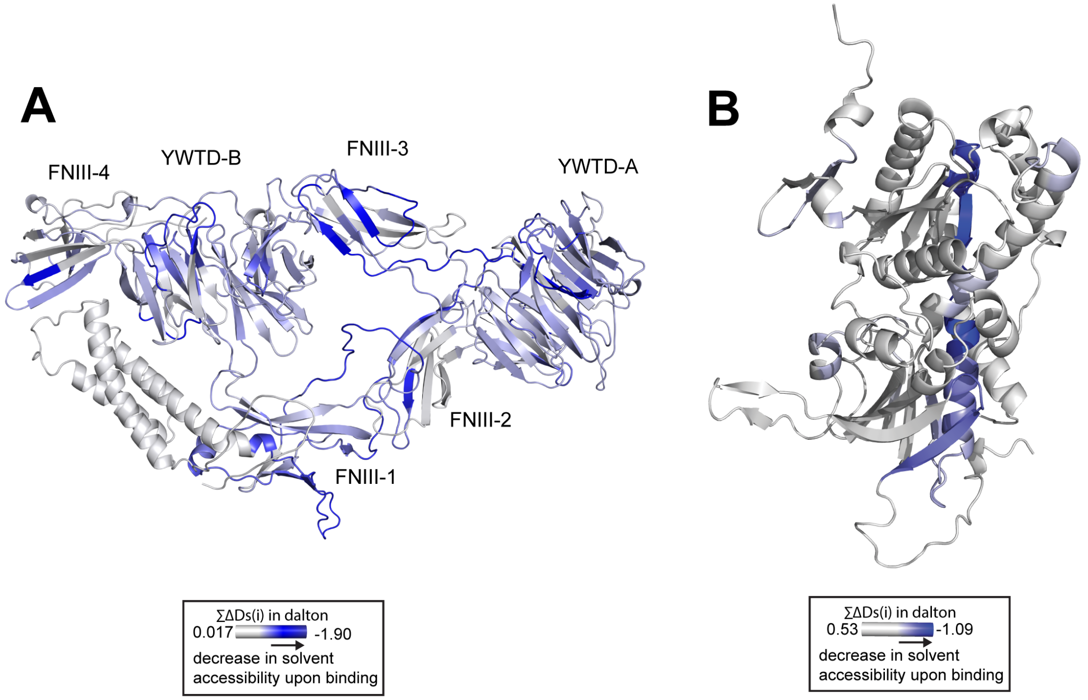

Supplementary Fig. 8 | HDX-MS results mapped onto dROS1 and BOSS structures

The ΣΔDs(i) value of each amino acid was assigned using DynamX and Pymol as described previously[4]. The ΣΔDs(i) values were color-coded and mapped onto AlphaFold predicted structure of dROS1 (A) or BOSS (B) to aid visualization of possible binding regions. The number on the left and right sides of the colored bar indicates the maximum and minimum ΣΔDs(i) values, respectively. Possible binding regions in dROS1 (A) and BOSS (B) have negative ΣΔDs(i) values (deuterium uptake of the complex-free ligand) and are colored blue on the structures. For the list of the ΣΔDs(i) value at each amino acid, see *The Source data file*.

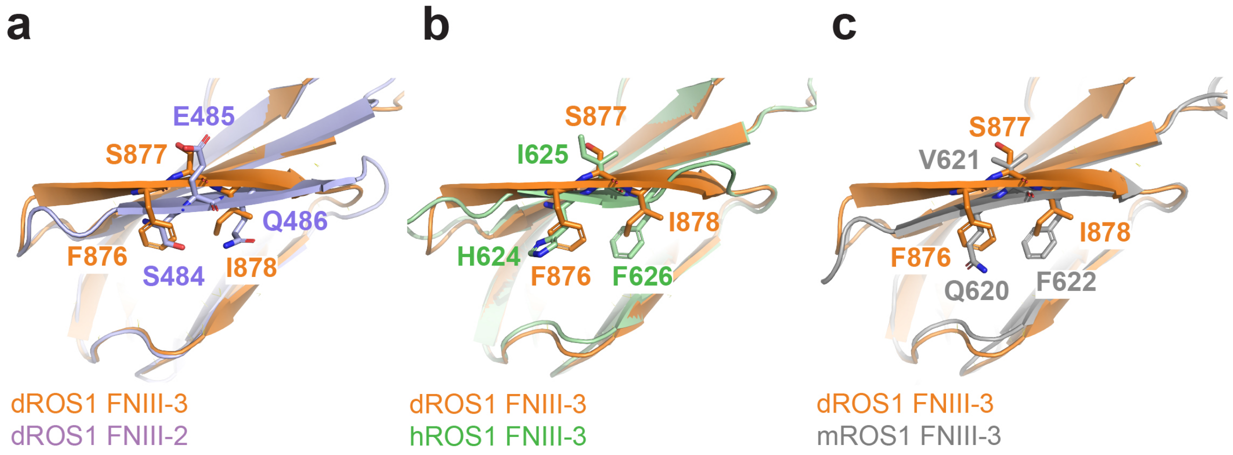

Supplementary Fig. 9 | Structural analysis of dROS1 binding epitope. a, Overlay of dROS1 FNIII-3 with dROS1 FNIII-2. **b,** hROS1 (human ROS1) FNIII-3, and **c,** mROS1 (mouse ROS1) FNIII-3. The hydrophobic residues “FSI” in dROS1’s FNIII-3 is not conserved in FNIII-2 (SEQ). In addition, the corresponding residue of F876 is not hydrophobic in both hROS1 and mROS1.

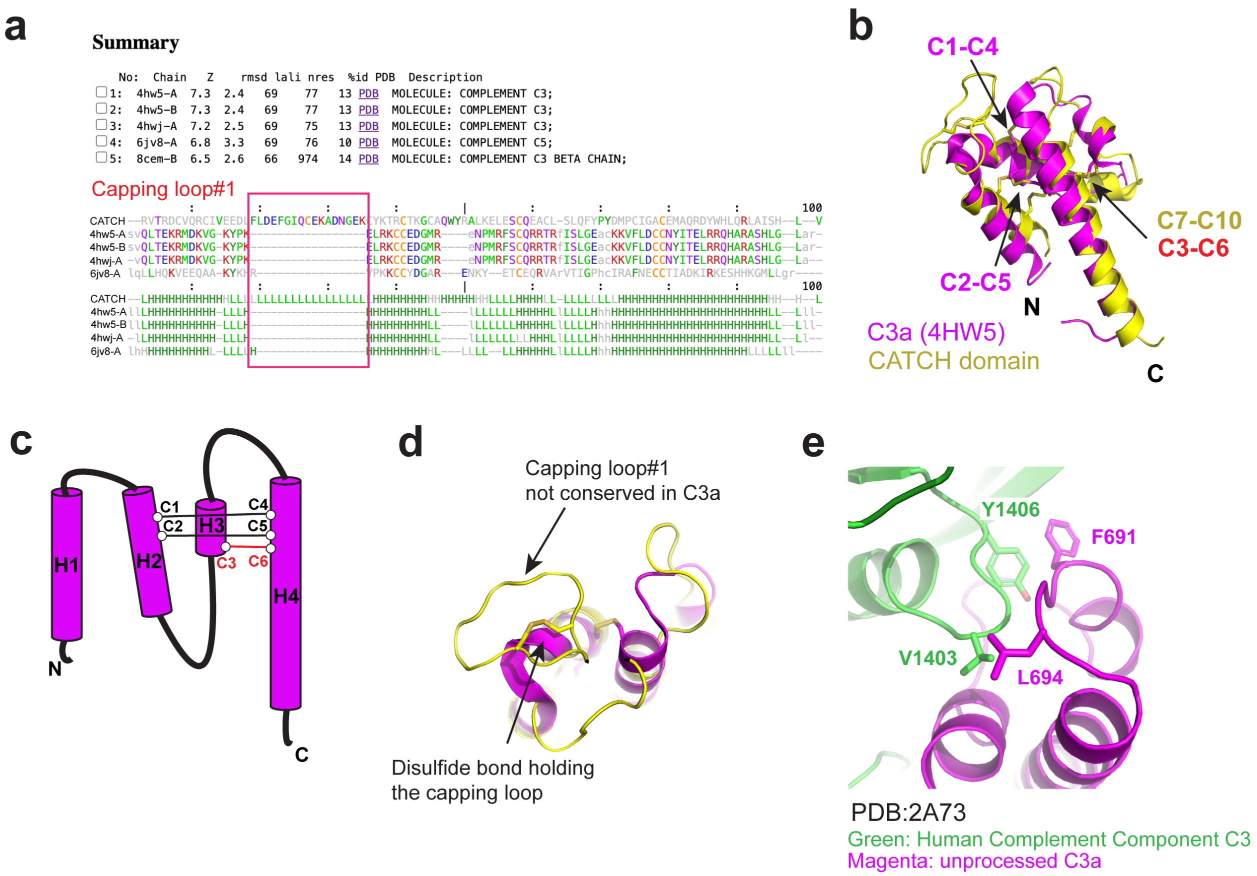

Supplementary Fig. 10 | Structural alignment search of the CATCH domain. a, Result from 3D alignment search using the Dali server[1,2]. The top candidate was Complement component protein C3a. Secondary structure alignment of the CATCH domain with the top 4 candidates shown. **b,** 3D alignment of CATCH domain with C3a (PDB: 4hw5, chain A)[5] (RMSD = 2.4). **c,** Topology and disulfide bonding illustration of C3a. The protein C3a has 3 disulfide bonds, with C1-C4 and C2-C5 connecting helix-2 (H2) and helix-4 (H4), and C3-C6 connecting H3 and H4. Note that C3-C6 colored in red is located at the same position as C7-C10 in dROS1's CATCH

domain. **d,** Capping loop 1 and the disulfide bond holding the capping loop are unique features of dROS1's CATCH domain. **e,** In the unprocessed C3 complement protein structure (PDB: 2A73)[6], the loop connecting C3a's H3 and H4 (equivalent to capping loop 2 in dROS1's CATCH domain) are interacting with the rest of the protein, similar to the interaction of capping loop 2 of CATCH with dROS1 FNIII-4.

**Protein**	dROS1	
PBD ID	9BHX	
**Data Collection**		
Microscope	FEI Titan Krios	
Magnification	105K	
Voltage (ke$^-$V)	300	
Electron exposure (e$^-$/ Å $^{-2}$)	50	
Detector	Gatan K3	
Pixel size (Å/pixel)	0.825	
Defocus range	-0.8 to -2.0	
Final number of particles for reconstruction	144,311	388,364
Symmetry imposed	C1	C1
Box size (pixels)	768	768
Map resolution (Å)	3.94	3.74
**Refinement**		
Number of non-H atoms	8376	
Protein residues	1048	
Molprobity score	2.26	
Clashscore	20.21	
Rotamer outlier (%)	0.00	
r.m.s.d. values		
Bond length (Å)	0.013	
Bond angles (Å)	1.331	
Ramachandran analysis		
Favored (%)	92.61	
Allowed (%)	6.91	
Outliers (%)	0.48	

Supplementary Table 1 | CryoEM data collection and refinement statistics of dROS1 ECR.

HDX-MS statistics	dROS1:BOSS	dROS1	BOSS:dROS1	BOSS
# of peptides	296	382	226	277
Peptide Coverage (%)	94.7	97.7	97.2	96.8
Ave peptide length/Redundancy	12.1/3.7	12.0/3.9	10.2/3.3	10.2/4.2
Replicates (n)	2	2	2	2
Repeatability (Ave SD, Da)	0.1766 Da	0.1687 Da	0.1364 Da	0.1304 Da
Significant difference in HDX[7]	0.7746 Da		0.5409 Da	

The significance at 90% confidence limit was calculated using the average SD of all analyzed peptides based on Houde et al.[7].

Supplementary Table 2 | HDX-MS statistics.

Supplementary References:

1 Holm, L. Dali server: structural unification of protein families. *Nucleic Acids Res* **50**, W210-W215, doi:10.1093/nar/gkac387 (2022).

2 Holm, L., Laiho, A., Toronen, P. & Salgado, M. DALI shines a light on remote homologs: One hundred discoveries. *Protein Sci* **32**, e4519, doi:10.1002/pro.4519 (2023).

3 Lin, S. *et al*. Structures of G(i)-bound metabotropic glutamate receptors mGlu2 and mGlu4. *Nature* **594**, 583-588, doi:10.1038/s41586-021-03495-2 (2021).

4 van Alderwerelt van Rosenburgh, I. K. *et al*. Biochemical and structural basis for differential inhibitor sensitivity of EGFR with distinct exon 19 mutations. *Nat Commun* **13**, 6791, doi:10.1038/s41467-022-34398-z (2022).

5 Bajic, G., Yatime, L., Klos, A. & Andersen, G. R. Human C3a and C3a desArg anaphylatoxins have conserved structures, in contrast to C5a and C5a desArg. *Protein Sci* **22**, 204-212, doi:10.1002/pro.2200 (2013).

6 Janssen, B. J. *et al*. Structures of complement component C3 provide insights into the function and evolution of immunity. *Nature* **437**, 505-511, doi:10.1038/nature04005 (2005).

7 Houde, D., Berkowitz, S. A. & Engen, J. R. The utility of hydrogen/deuterium exchange mass spectrometry in biopharmaceutical comparability studies. *J Pharm Sci* **100**, 2071-2086, doi:10.1002/jps.22432 (2011).
