# Supplementary material for: Structural basis for the interaction between the Drosophila RTK Sevenless (dROS1) and the GPCR BOSS
Source: Nat Commun. 2025 Jan 18;16:808. doi: 10.1038/s41467-025-55943-6 (PMC11743138; doi:10.1038/s41467-025-55943-6)
Supplement: Supplementary file 2 — Reporting Summary [file 41467_2025_55943_MOESM2_ESM.pdf]

nature portfolio

Corresponding author(s): Daryl E. Klein

Last updated by author(s): Oct 7, 2024

Reporting Summary

Nature Portfolio wishes to improve the reproducibility of the work that we publish. This form provides structure for consistency and transparency in reporting. For further information on Nature Portfolio policies, see our Editorial Policies and the Editorial Policy Checklist.

Statistics

For all statistical analyses, confirm that the following items are present in the figure legend, table legend, main text, or Methods section.

n/a	Confirmed	
☐	☒	The exact sample size (*n*) for each experimental group/condition, given as a discrete number and unit of measurement
☐	☒	A statement on whether measurements were taken from distinct samples or whether the same sample was measured repeatedly
☐	☒	The statistical test(s) used AND whether they are one- or two-sided<br>*Only common tests should be described solely by name; describe more complex techniques in the Methods section.*
☒	☐	A description of all covariates tested
☒	☐	A description of any assumptions or corrections, such as tests of normality and adjustment for multiple comparisons
☐	☒	A full description of the statistical parameters including central tendency (e.g. means) or other basic estimates (e.g. regression coefficient) AND variation (e.g. standard deviation) or associated estimates of uncertainty (e.g. confidence intervals)
☐	☒	For null hypothesis testing, the test statistic (e.g. *F*, *t*, *r*) with confidence intervals, effect sizes, degrees of freedom and *P* value noted<br>*Give P values as exact values whenever suitable.*
☒	☐	For Bayesian analysis, information on the choice of priors and Markov chain Monte Carlo settings
☒	☐	For hierarchical and complex designs, identification of the appropriate level for tests and full reporting of outcomes
☒	☐	Estimates of effect sizes (e.g. Cohen's *d*, Pearson's *r*), indicating how they were calculated

Our web collection on statistics for biologists contains articles on many of the points above.

Software and code

Policy information about availability of computer code

Data collection	CryoEM data were collected from the Titan Krios located at Yale CryoEM Center at West Campus. Binding data were collected on BIAcore T200. Coomassie-stained gels were imaged using Bio-Rad ChemiDoc MP imaging system. HDX-MS data were collected using Synapt G2-Si (Water) mass spectrometer.
Data analysis	CryoEM data were processed using CryoSPARC v4.4.1 and the final model was refined using Phenix v1.21-5207 and Coot v0.9.6 from SBGrid v2.2.16. Final structure was validated with MolProbity and wwPDB servers. Coomassie-stained gel images were analyzed using ImageJ v6.0.1. Binding data were analyzed using Prism 9. For HDX-MS dataset, ProteinLynx Global Server 3.03 (Waters) was used to sequence each peptic peptide, and the deuterium uptake of peptic peptides was evaluated using DynamX 3.0 (Waters). Structural figures were generated using PyMol v2.5.4 and ChimeraX v1.6.1.

For manuscripts utilizing custom algorithms or software that are central to the research but not yet described in published literature, software must be made available to editors and reviewers. We strongly encourage code deposition in a community repository (e.g. GitHub). See the Nature Portfolio guidelines for submitting code & software for further information.

Data

Policy information about availability of data

All manuscripts must include a data availability statement. This statement should provide the following information, where applicable:

- Accession codes, unique identifiers, or web links for publicly available datasets
- A description of any restrictions on data availability
- For clinical datasets or third party data, please ensure that the statement adheres to our policy

The refined structural model and the CryoEM map, half maps and mask have been deposited under PDB accession code 9BHX (protein sevenless).and in the EMDB with accession codes EMD-44556.

Research involving human participants, their data, or biological material

Policy information about studies with human participants or human data. See also policy information about sex, gender (identity/presentation), and sexual orientation and race, ethnicity and racism.

Reporting on sex and gender	*Use the terms sex (biological attribute) and gender (shaped by social and cultural circumstances) carefully in order to avoid confusing both terms. Indicate if findings apply to only one sex or gender; describe whether sex and gender were considered in study design; whether sex and/or gender was determined based on self-reporting or assigned and methods used. Provide in the source data disaggregated sex and gender data, where this information has been collected, and if consent has been obtained for sharing of individual-level data; provide overall numbers in this Reporting Summary. Please state if this information has not been collected. Report sex- and gender-based analyses where performed, justify reasons for lack of sex- and gender-based analysis.*
Reporting on race, ethnicity, or other socially relevant groupings	*Please specify the socially constructed or socially relevant categorization variable(s) used in your manuscript and explain why they were used. Please note that such variables should not be used as proxies for other socially constructed/relevant variables (for example, race or ethnicity should not be used as a proxy for socioeconomic status). Provide clear definitions of the relevant terms used, how they were provided (by the participants/respondents, the researchers, or third parties), and the method(s) used to classify people into the different categories (e.g. self-report, census or administrative data, social media data, etc.) Please provide details about how you controlled for confounding variables in your analyses.*
Population characteristics	*Describe the covariate-relevant population characteristics of the human research participants (e.g. age, genotypic information, past and current diagnosis and treatment categories). If you filled out the behavioural & social sciences study design questions and have nothing to add here, write "See above."*
Recruitment	*Describe how participants were recruited. Outline any potential self-selection bias or other biases that may be present and how these are likely to impact results.*
Ethics oversight	*Identify the organization(s) that approved the study protocol.*

Note that full information on the approval of the study protocol must also be provided in the manuscript.

Field-specific reporting

Please select the one below that is the best fit for your research. If you are not sure, read the appropriate sections before making your selection.

☒ Life sciences ☐ Behavioural & social sciences ☐ Ecological, evolutionary & environmental sciences

For a reference copy of the document with all sections, see nature.com/documents/nr-reporting-summary-flat.pdf

Life sciences study design

All studies must disclose on these points even when the disclosure is negative.

Sample size	Predetermination of sample size was not performed in this study. Independent biological repeats of experiments were performed. HDX-MS were repeated with different batches of protein.
Data exclusions	No data was excluded.
Replication	Binding assays were performed with at least 3 biological repeats from different batches of proteins when possible, except for the dROS1 SEQ mutant and BOSS wild type in BOSS DEQ mutant experiment in which only 2 biological repeats were performed. HDX-MS experiments were repeated independently from different preparations of protein (n=2), and 3 technical repeats foe each n experiment were performed.
Randomization	not relevant for this study
Blinding	Blinding was not applicable to this work because this type of study does not use group allocation.

Reporting for specific materials, systems and methods

We require information from authors about some types of materials, experimental systems and methods used in many studies. Here, indicate whether each material, system or method listed is relevant to your study. If you are not sure if a list item applies to your research, read the appropriate section before selecting a response.

Materials & experimental systems

n/a	Involved in the study
☐	☒ Antibodies
☐	☒ Eukaryotic cell lines
☒	☐ Palaeontology and archaeology
☒	☐ Animals and other organisms
☒	☐ Clinical data
☒	☐ Dual use research of concern
☒	☐ Plants

Methods

n/a	Involved in the study
☒	☐ ChIP-seq
☒	☐ Flow cytometry
☒	☐ MRI-based neuroimaging

Antibodies

Antibodies used	Penta-his antibody (Qiagen, Cat. No. 34660),anti-ALK antibody (ALK (D5F3®) XP® Rabbit mAb, Cell Signaling Technology, no. 3633), anti-phospho ALK (Y1507) antibody (Phospho-ALK (Tyr1507) (D6F1V) Rabbit mAb , Cell Signaling Technology, no. 14678),The HRP-linked anti-rabbit IgG (Cell Signaling Technology, no. 7074S), The HRP-linked anti-mouse IgG (Abcam, ab97046)
Validation	Penta·His Antibody, BSA-free is used to detect recombinant protein carrying His tags. Highly specific detection of C-terminal, N-terminal and internal His tags, specific detection of RGS·His epitopes and HHHHH epitopes. ALK (D5F3®) XP® Rabbit mAb detects endogenous levels of total ALK protein as well as ALK fusion proteins, such as EML4-ALK variants and NPM-ALK, even at low levels. This antibody does not cross-react with other family members. Species Reactivity:Human. Phospho-ALK (Tyr1507) (D6F1V) Rabbit mAb recognizes endogenous levels of ALK protein only when phosphorylated at Tyr1507 (equivalent to Tyr567 of NPM-ALK). Species Reactivity:Human. Species predicted to react based on 100% sequence homology: Mouse, Rat.

Eukaryotic cell lines

Policy information about cell lines and Sex and Gender in Research

Cell line source(s)	NIH 3T3 cell line (ATCC CRL-1658) was used to generate a stable cell line
Authentication	No authentication after use
Mycoplasma contamination	The cell line tested negative for mycoplasma contamination
Commonly misidentified lines (See ICLAC register)	No common misidentified lines

Plants

Seed stocks	*Report on the source of all seed stocks or other plant material used. If applicable, state the seed stock centre and catalogue number. If plant specimens were collected from the field, describe the collection location, date and sampling procedures.*
Novel plant genotypes	*Describe the methods by which all novel plant genotypes were produced. This includes those generated by transgenic approaches, gene editing, chemical/radiation-based mutagenesis and hybridization. For transgenic lines, describe the transformation method, the number of independent lines analyzed and the generation upon which experiments were performed. For gene-edited lines, describe the editor used, the endogenous sequence targeted for editing, the targeting guide RNA sequence (if applicable) and how the editor was applied.*
Authentication	*Describe any authentication procedures for each seed stock used or novel genotype generated. Describe any experiments used to assess the effect of a mutation and, where applicable, how potential secondary effects (e.g. second site T-DNA insertions, mosiacism, off-target gene editing) were examined.*
